# Supplementary material for: Approach to standardized material characterization of the human lumbopelvic system—Specification, preparation and storage
Source: PLoS One. 2023 Aug 3;18(8):e0289482. doi: 10.1371/journal.pone.0289482 (PMC10399898; doi:10.1371/journal.pone.0289482)
Supplement: S1 File — Including visual harvesting protocol for the lumbopelvic system, assessment code scheme as well as labels for storage boxes. (PDF) [file pone.0289482.s001.pdf]

Standard Operating Procedure - Harvesting

Soft tissue harvesting list (s)

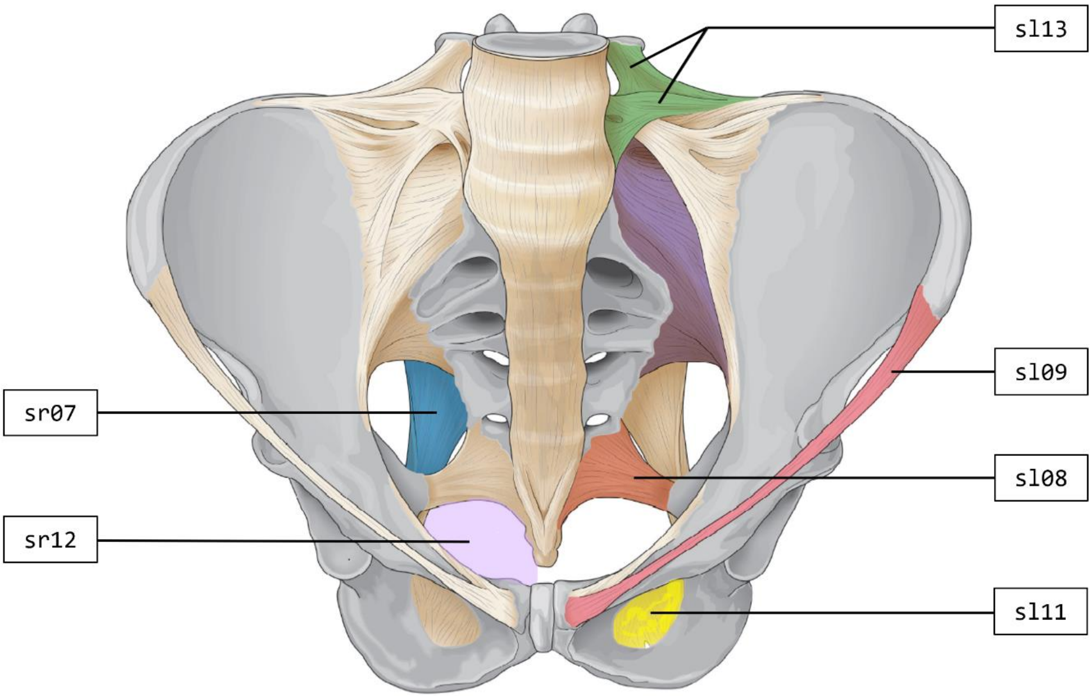

Figure 1: Pelvis ventral view (Modified from Kurz S et al., Biomechanik des Beckens Orthopädie und Unfallchirurgie up2date 2017; 12(01): 27-44 DOI: 10.1055/s-0042-109409 © Georg Thieme Verlag KG Stuttgart · New York) *

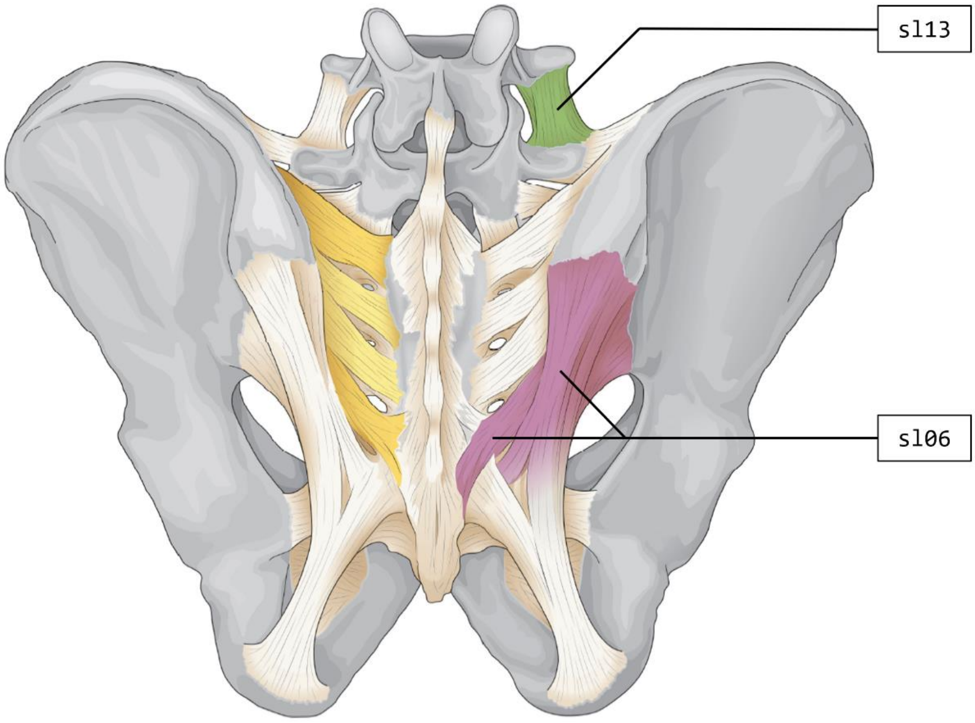

Figure 2: Pelvis dorsal view (Modified from Kurz S et al., Biomechanik des Beckens Orthopädie und Unfallchirurgie up2date 2017; 12(01): 27-44 DOI: 10.1055/s-0042-109409 © Georg Thieme Verlag KG Stuttgart · New York) *

** This figure is excluded from secondary publications under open access licenses. Any further use must be requested again and separately from permission@thieme.de.*

Harvesting protocol soft tissue (s)

No.	ID	Designation	Donor no.:	Date:
			T/°C:	*RH/%:*
			**Notes**	
1	sl01	Fascia glutea L		
2	sr01	Fascia glutea R		
3	sl02	Fascia thoracolumbalis lamina superficalis L		
4	sr02	Fascia thoracolumbalis lamina superficalis R		
5	sl03	Fascia thoracolumbalis lamina profunda L		
6	sr03	Fascia thoracolumbalis lamina profunda R		
7	sl04	Fascia crescent L		
8	sr04	Fascia crescent R		
9	sl05	Ligamenta sacroiliaca posteriora L		
10	sr05	Ligamenta sacroiliaca posteriora R		
11	sl06	Ligamentum sacroiliacum posterior longum L		
12	sr06	Ligamentum sacroiliacum posterior longum R		
13	sl07	Ligamentum sacrotuberale L		
14	sr07	Ligamentum sacrotuberale R		
15	sl08	Ligamentum sacrospinale L		
16	sr08	Ligamentum sacrospinale R		
17	sl09	Ligamentum inguinale L		
18	sr09	Ligamentum inguinale R		
19	sl10	Ligamentum pectineum L		
20	sr10	Ligamentum pectineum R		
21	sl11	Membrana obturatoria L		
22	sr11	Membrana obturatoria R		
23	sl12	Fascia endopelvina L		
24	sr12	Fascia endopelvina L		
25	sl13	Ligamentum iliolumbale L		
26	sr13	Ligamentum iliolumbale R		

Segmentation definition of trabecular bone (t) and cortical bone (c)

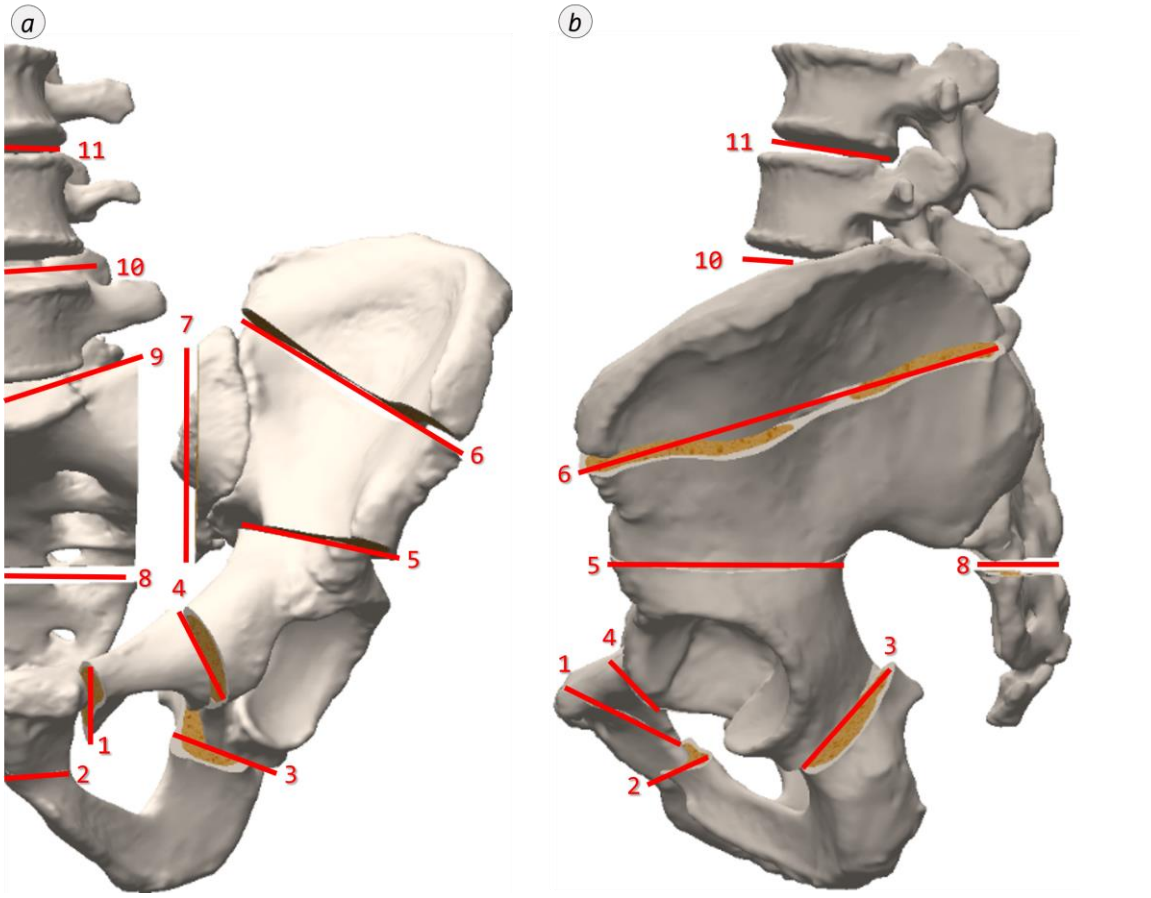

Figure 3: Definition of the segmentation cut sequence (*a*: ventral view, *b*: lateral view).

No.	Sectioning plane	Definition
1	Lower parasymphyseal	Tuberculum pubicum, parallel to Symphysis pubica
2	Ramus ossis ischia	Ramus inferior ossis pubis to Symphysis pubica, parallel to the inferior end of the Symphysis pubica
3	Upper parasymphyseal	Foramen obturatum through Corpus ossis ischii, parallel to the inferior end of the Acetabulum, superior to the Spina ischiadica
4	Ramus superior ossis pubis	Corpus ossis pubis, from Tuberculum between Corpus and Ramus superior ossis pubis, normal to Ramus superior ossis pubis
5	Supraacetabular	Spina iliaca anterior inferior to Incisura ischiadica major, through Sulcus supraacetabularis
6	Ala ossis ilium	Spina iliaca anterior superior to Spina iliaca posterior superior, normal to Fossa iliaca
7	Os sacrum, Pars lateralis	Between Crista sacralis lateralis and Articulatio sacroiliaca, parallel to Sagittal plane
8	Os sacrum	Lineae transversae between Corpus vertebrae sacralis 2 and 3
9	Basis ossis sacri	Separating Corpus vertebrae 5 from Os sacrum, through Discus intervertebralis
10	Intervertebralis 4/5	Separating Corpus vertebrae 4 and 5, through Discus intervertebralis
11	Intervertebralis 3/4	Separating Corpus vertebrae 3 and 4, through Discus intervertebralis

Incisions that are present on both sides must be made one after the other.

Area classification of trabecular bone (t) and cortical bone (c)

Declaration: area|segment

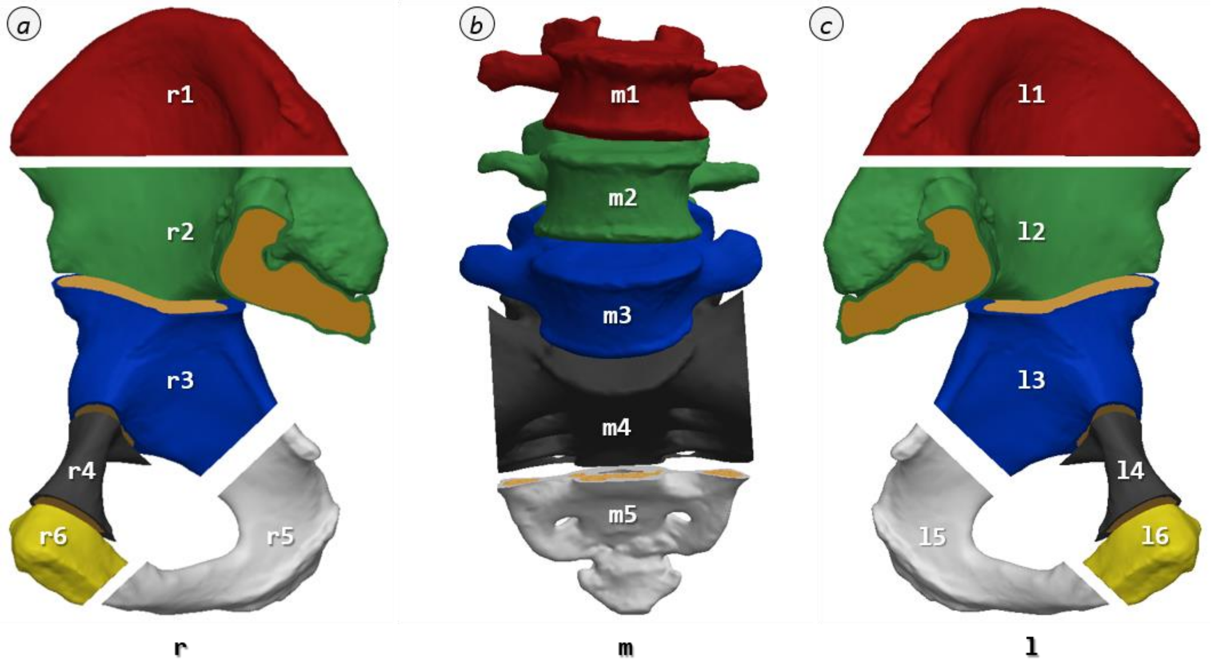

Figure 4: Segment definition (*a*: lateral view of the segment group right (**r**), *b*: ventral view of the medial segment group (**m**), *c*: lateral view of the segment group left (**l**)).

Harvesting point designation cancellous bone (s) and cortical bone (c)

Harvesting point	Description
r11 / l11	Ala ossis ilii superior R/L anterior
r12 / l12	Ala ossis ilii superior R/L posterior
r13 / l13	Ala ossis ilii superior R/L anterior superior
r21 / l21	Ala ossis ilii inferior R/L anterior
r22 / l22	Ala ossis ilii inferior R/L posterior
r31 / l31	Corpus ossis ilii supraacetabular R/L
r32 / l32	Corpus ossis ischii postacetabular R/L
r41 / l41	Ramus superior ossis pubis R/L
r51 / l51	Ramus ossis ischii R/L
m21	Corpus vertebrae lumbales 4
m31	Corpus vertebrae lumbales 5
m41	Corpus vertebrae sacrales 1

Visual harvesting protocol trabecular bone (t) and cortical bone (c)

Declaration: specimen type|segment|subsegment|harvesting location|harvesting point

Example: trabecular bone|r|2|1|y

Resulting key: `tr21y`

Cortical bone: a=inner, b=outer

Trabecular bone: Designation according to test direction

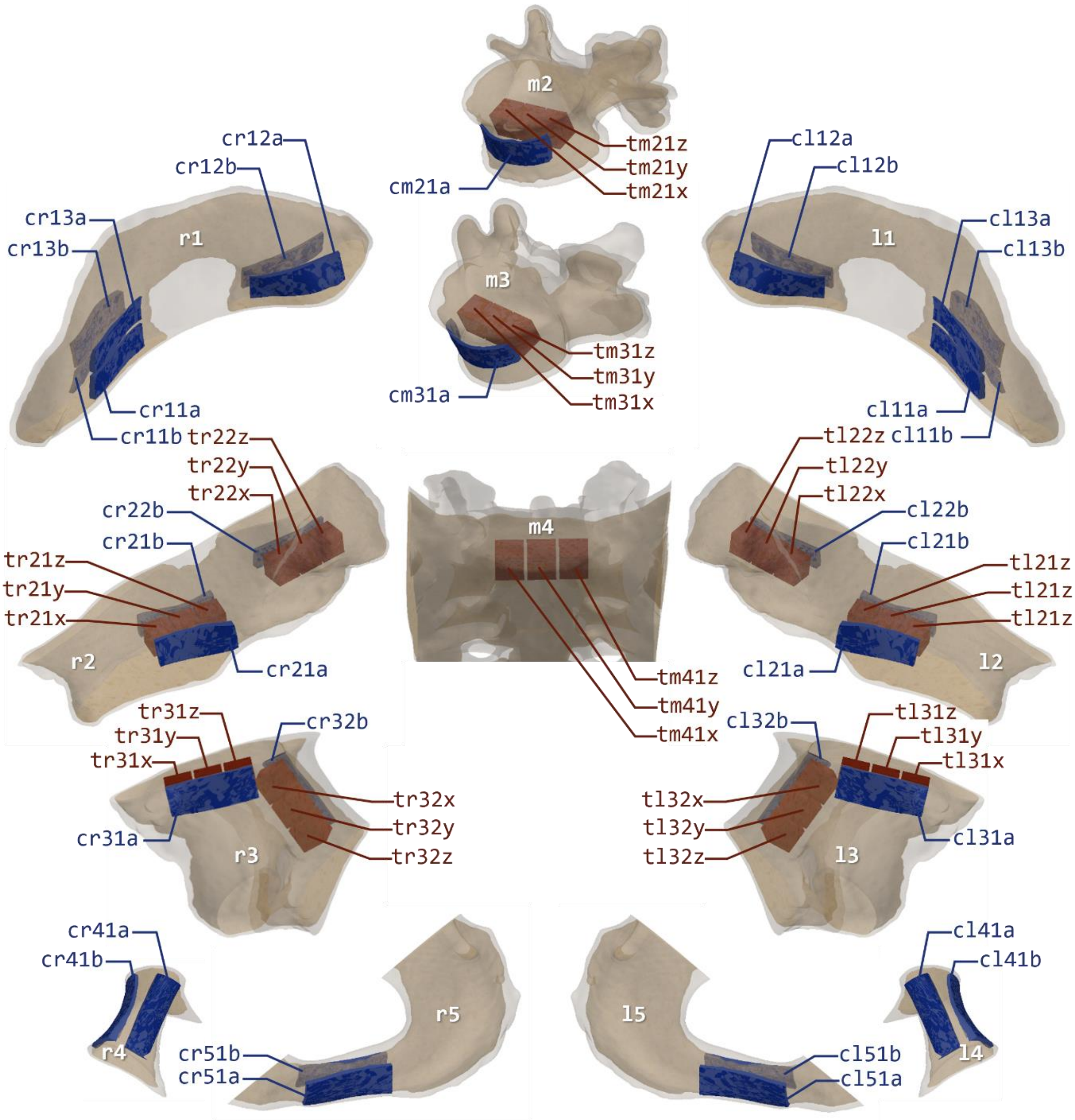

Figure 5: Definition of harvesting points

cm21a and cm31a should only be taken if the curvature is very moderate!

Storage and testing specifications trabecular bone (t) and cortical bone (c)

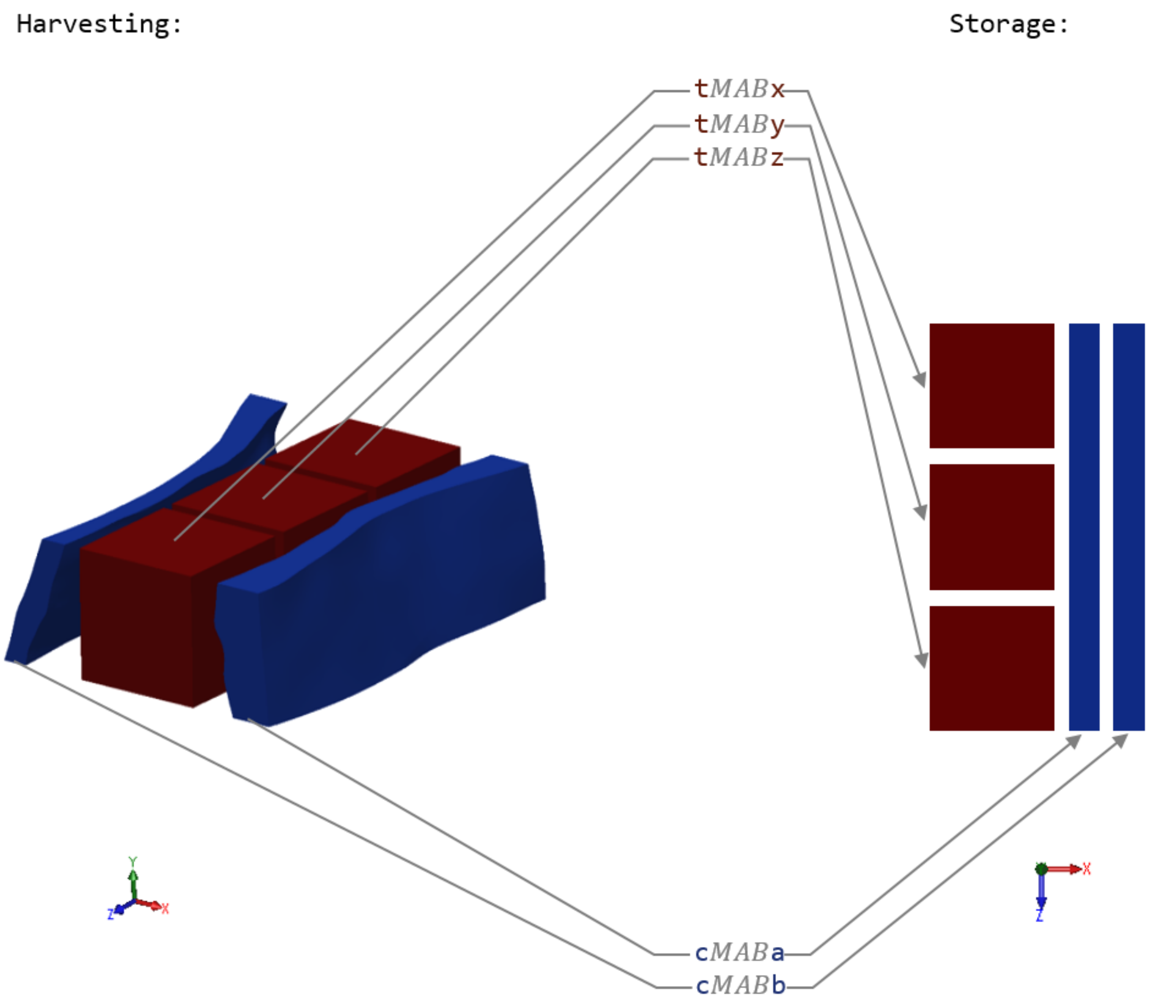

Figure 6: Storage specifications

Testing:

tMABx in x-direction
tMABy in y-direction
tMABz in z-direction
Key recommended for digital data processing:

{ORCID} _{Preparation location Key}_{Donor ID}_{Anatomical Region Key}_{Specimen Key}

Example: Marc Gebhardt, Leipzig-University Leipzig-Anatomy, local donor ID 67-17, cortical bone specimen from Ramus superior ossis pubis R proximal

Resulting key: 0000-0001-8378-3108_LEIULANA_67-17_LuPeCo_cr41a

Harvesting protocol trabecular bone (t)

No.	ID	Storage-box	Density specimen	Donor no.:	Date:
				T/°C:	RH/%:
				Notes	
01	tr21x				
02	tr21y	1			
03	tr21z				
04	tr22x				
05	tr22y	2			
06	tr22z				
07	tl21x				
08	tl21y	3			
09	tl21z				
10	tl22x				
11	tl22y	4			
12	tl22z				
13	tr31x				
14	tr31y	5			
15	tr31z				
16	tr32x				
17	tr32y	6			
18	tr32z				
19	tl31x				
20	tl31y	7			
21	tl31z				
22	tl32x				
23	tl32y	8			
24	tl32z				
25	tm21x				
26	tm21y	9			
27	tm21z				
28	tm31x				
29	tm31y	10			
30	tm31z				
31	tm41x				
32	tm41y	11			
33	tm41z				

Harvesting protocol cortical bone (c)

No.	ID	Storage-box	Density specimen	Donor no.:	Date:
				T/°C:	*RH/%:*
				Notes	
01	cr11a	A			
02	cr11b				
03	cr12a	A			
04	cr12b				
05	cr13a	B			
06	cr13b				
07	cl11a	B			
08	cl11b				
09	cl12a	C			
10	cl12b				
11	cl13a	C			
12	cl13b				
13	cr21a	1			
14	cr21b				
15	cr22b	2			
16	cl21a	3			
17	cl21b				
18	cl22b	4			
19	cr31a	5			
20	cr32b	6			
21	cl31a	7			
22	cl32b	8			
23	cr41a	D			
24	cr41b				
25	cl41a	D			
26	cl41b				
27	cr51a	E			
28	cr51b				
29	cl51a	E			
30	cl51b				
31	cm21a	9			
32	cm31a	10			

Annex 1: Assessment code scheme

This biomechanical assessment scheme was developed to document any deviations or peculiarities within the entire procedure. It allows the evaluation of individual specimens concerning different procedure steps, assessment types and the strength of their occurrence. Thus, errors in the procedure in general as well as the reliability of the results can be quantified based on defined criteria and evaluated retrospectively. Table 1 shows this very assessment scheme.

Table 1: Biomechanical-assessment-code-scheme (Version 3.2)

**Procedure Step**			**Assessment Type**				**Strength**	
	**Description**	**Key x**	**Description**	**Key y**	**Description**	**Key y**	**Description**	**Key z**
Acqui-sition	Pre-dissection	A	Abnormal	00	Mass	11	*Not*	*0*
	Dissection	B	Absence	01	Moisture	12	Slight	1
	Storage	C	Damaged	02	Designation	13	Medium	2
Test	Testing	D	Deflection	03	Optical	14	Complete	3
	Measurement	E	Density	04	Orientation	15		
Evalu-ation	Input	F	Displacement	05	Scheme	16		
	Statistics	G	Force	06	Temperature	17		
Combined Code: {Key x}{Key y}.{Key z}			Geometrical	07	Timing	18		
			Histological	08	Strain	19		
			Impure	09	…	…		
			Localization	10				

The above-shown scheme leads to a combined code composed out of three partial keys. The first key describes the procedural step in which the assessment takes place; e.g. if the assessment is performed during the specimen dissection, *Key x = B*. The second key indicates the type of assessment, e.g. a damaged specimen is characterized by *Key y = 02*. Key three indicates the strength of the occurrence. For Example, a medium damaged specimen will lead to *Key y.Key z = 02.2*. Thus, the combined assessment code for this example would be *B02.2*. Other examples of coding an assessment are shown in Table 2.

Table 2: Application examples of the biomechanical-assessment-code (Version 3.2) generation

**Assessment**	**Biomechanical-assessment-code**
Specimen severely damaged during preliminary preparation and therefore not suitable for harvesting	A02.3, B01.3
Melanoma within trabecular bone specimen location, completely damaged during dissection	A00.3, B02.3
Specimen perhaps rotated	B15.2
Specimen stored outside the box, probably dried out	C12.2
Touching of test device after maximum loading	D03.1
Touching of test device before maximum loading	D03.2
Slippage of the specimen during the test	D03.3
Aborting the test by reaching the force limit	D06.3
Medium LVDT (linear variable displacement transformer) error	E05.2
Optical displacement measuring inaccurate due to low lighting	E14.2
Corrupted measurement data	F02.3
Remarkable deviation of the material data in contrast to comparable samples	G00.2

1	tr2	1z, 1y, 1x	cr21a	cr21b		
2	tr2	2z, 2y, 2x		cr22b		
3	tl2	1z, 1y, 1x	cl21a	cl21b		
4	tl2	2z, 2y, 2x		cl22b		
5	tr3	1z, 1y, 1x	cr31a			
6	tr3	2z, 2y, 2x		cr32b		
7	tl3	1z, 1y, 1x	cl31a			
8	tl3	2z, 2y, 2x		cl32b		
9	tm2	1z, 1y, 1x	cm21a			
10	tm3	1z, 1y, 1x	cm31a			
11	tm4	1z, 1y, 1x				
A			cr11a	cr11b	cr12a	cr12b
B			cr13a	cr13b	cl11a	cl11b
C			cl12a	cl12b	cl13a	cl13b
D			cr41a	cr41b	cl41a	cl41b
E			cr51a	cr51b	cl51a	cl51b

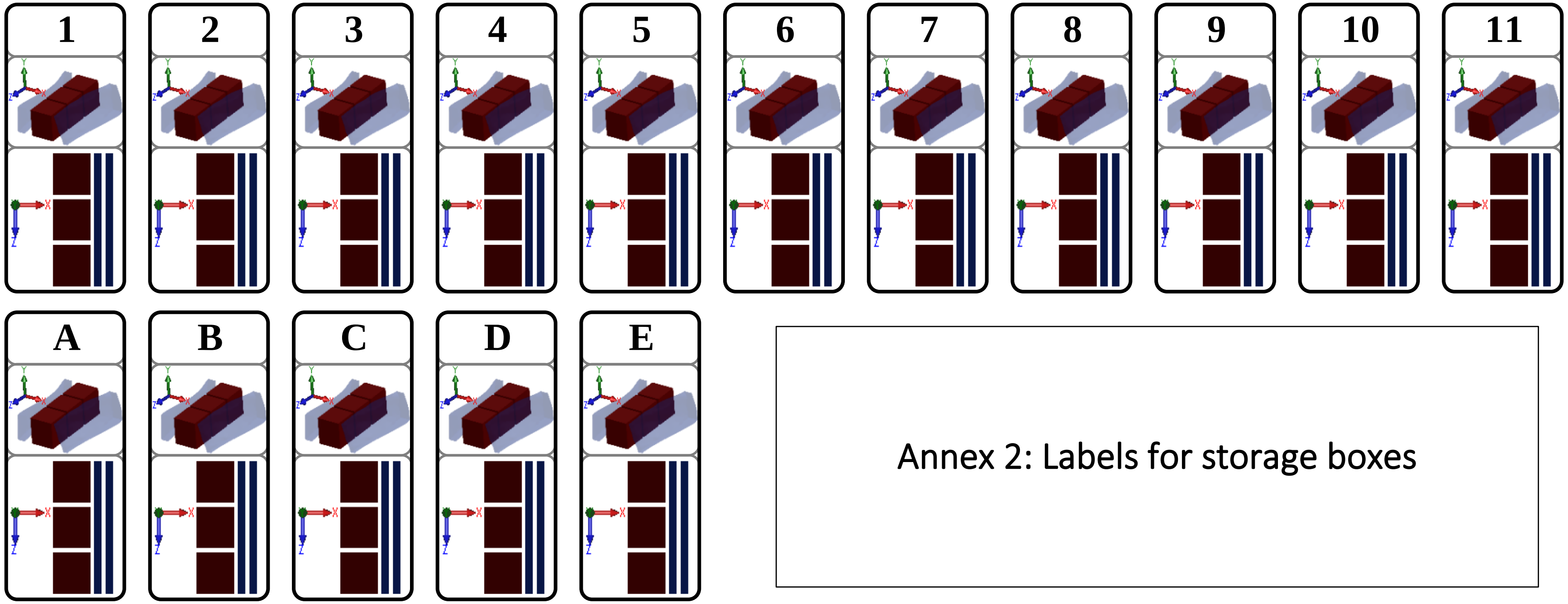

Annex 2: Labels for storage boxes
