# Supplementary material for: Approach to standardized material characterization of the human lumbopelvic system—Specification, preparation and storage
Source: PLoS One. 2023 Aug 3;18(8):e0289482. doi: 10.1371/journal.pone.0289482 (PMC10399898; doi:10.1371/journal.pone.0289482)
Supplement: S2 File — Including 3D models and 3D-pdf overviews of preparation auxiliaries and storage boxes. (ZIP) [file pone.0289482.s002.zip › Preparation/Saw_Guide/Saw_guide.pdf]

Saw guide for adjustable bone cutting

UNIVERSITÄT LEIPZIG
Medizinische Fakultät

ZESBO
Zentrum zur Erforschung der Stütz- und Bewegungsorgane

Title	Saw guide
Subject	Biomechanics-Preparation
Revision	2021-03-18-001
Author	Kurz, Sascha

Notes

Supplementary material to "Approach to Standardized Material Characterization of the Human Lumbopelvic System".

Saw guide with +/- 45° angle adjustment and triple adjustable 10 mm cutting intervals.

Manufacturing via FDM. Tested with following settings:
- Nozzle = 0.4 mm
- Filament material = PLA
- Resolution = 0.2 mm

Additional material:
- 2 Socket Head Cap Screw ISO 4762-M6x20
- 2 Hexagon Nut ISO 4032-M6

Supported by:

Federal Ministry for Economic Affairs and Energy

on the basis of a decision by the German Bundestag

ZIM Zentrales Innovationsprogramm Mittelstand

CC BY
