# Supplementary material for: Approach to standardized material characterization of the human lumbopelvic system—Specification, preparation and storage
Source: PLoS One. 2023 Aug 3;18(8):e0289482. doi: 10.1371/journal.pone.0289482 (PMC10399898; doi:10.1371/journal.pone.0289482)
Supplement: S2 File — Including 3D models and 3D-pdf overviews of preparation auxiliaries and storage boxes. (ZIP) [file pone.0289482.s002.zip › Preparation/Saw_Jig-Trabecular_Cube/Saw_jig-Cube_10mm.pdf]

# Saw jig for cutting trabecular bone cubes

HTWK Leipzig University of Applied Sciences
Faculty of Civil Engineering

| | |
|---|---|
| Title | Saw jig - Cube 10mm |
| Subject | Biomechanics-Preparation |
| Revision | 2021-03-08-001 |
| Author | Gebhardt, Marc |

Notes

Supplementary material to "Approach to Standardized Material Characterization of the Human Lumbopelvic System".

Saw jig for cutting trabecular bone cubes with 10 mm edge length by using a band saw.

Manufacturing via FDM. Tested with following settings:
- Nozzle = 0.4 mm
- Filament material = PLA
- Resolution = 0.2 mm

Supported by:
Federal Ministry for Economic Affairs and Energy
on the basis of a decision by the German Bundestag

ZIM Zentrales Innovationsprogramm Mittelstand

CC BY
