# Supplementary material for: Approach to standardized material characterization of the human lumbopelvic system—Specification, preparation and storage
Source: PLoS One. 2023 Aug 3;18(8):e0289482. doi: 10.1371/journal.pone.0289482 (PMC10399898; doi:10.1371/journal.pone.0289482)
Supplement: S2 File — Including 3D models and 3D-pdf overviews of preparation auxiliaries and storage boxes. (ZIP) [file pone.0289482.s002.zip › Storage/Storage Box-Combined_Trabecular_and_Cortical_Bone/Storage_Box-Combined_Trabecular_Cortical_Bone.pdf]

Storage box for trabecular and cortical bone specimens

HTWK
Leipzig University
of Applied Sciences
Faculty of
Civil Engineering

Title	Storage Box - Combined bone specimens
Subject	Biomechanics-Storage
Revision	2021-03-08-001
Author	Gebhardt, Marc

Notes

Supplementary material to "Approach to Standardized Material Characterization of the Human Lumbopelvic System".

Storage box for trabecular and cortical bone specimens.
- Trabecular bone:
  - 3 cubes
  - 10 mm edge length
- Cortical bone:
  - 2 beams
  - 36 mm length and 10 mm width

Manufacturing via FDM. Tested with following settings:
- Nozzle = 0.4 mm
- Filament material = PLA
- Resolution = 0.2 mm

Labels available as annex of supplementary material "Standard Operating Procedure - Harvesting".

Supported by:
Federal Ministry
for Economic Affairs
and Energy
on the basis of a decision
by the German Bundestag

ZIM
Zentrales
Innovationsprogramm
Mittelstand

CC BY
