# Supplementary material for: Approach to standardized material characterization of the human lumbopelvic system—Specification, preparation and storage
Source: PLoS One. 2023 Aug 3;18(8):e0289482. doi: 10.1371/journal.pone.0289482 (PMC10399898; doi:10.1371/journal.pone.0289482)
Supplement: Attachment — Submitted filename: ASC-P1_2nd_response_point-by-point_2023-07-18.pdf [file pone.0289482.s005.pdf]

Response to Reviewers - Point-by-point

The authors would like to thank for the important and beneficial comments, all of them were considered. The manuscript has been revised accordingly and our responses can be found below (blue text).

Reviewer #1: To the authors

The reviewer has over 18 years of research experience in biomechanical experiments using cadavers. I read the authors' paper with great interest. It is mentioned that the paper provides a detailed description of the aspects that all researchers in the current field are concerned about. However, these aspects are already well known to other researchers in the related field and are widely practiced and acknowledged. Hence, it appears challenging to highly evaluate the academic value of these papers.

Thank you for your honest and direct assessment. We are aware that many scientists are familiar with the difficulties of working with cadavers and try to solve them to the best of their knowledge and belief. However, the respective ways are unfortunately often very different and make good work sometimes difficult to compare. Our effort was therefore to show a way as simple as possible to address these problems while still achieving comparable results. So that many can contribute with their research to a comparable data set.

Reviewer #2: To the authors

The paper looks really good. The previous revisions have been completed. I am not a statistician so I did not comment on the stats however, they looked good to me. Only additional revision I have is to add specific anatomical landmarks for the boney pelvis cuts. Some of the specific landmarks were listed on page 16, lines 184-189, but it would be nice to know where all of the cuts were made. Especially since it was mentioned on page 16, line 177 that a "plane was drawn between each two landmarks". Finally I though figure 9 was awesome!

Thank you very much for your constructive comments and encouraging words, we really appreciate it. For a better description of the sectioning planes we adjusted lines 176, 181, 184 and 186-188. Also we improved Fig 3 and added Table 1 for a clearer and more detailed description of the sectioning planes without overloading the text.
