# Supplementary material for: Approach to standardized material characterization of the human lumbopelvic system—Specification, preparation and storage
Source: PLoS One. 2023 Aug 3;18(8):e0289482. doi: 10.1371/journal.pone.0289482 (PMC10399898; doi:10.1371/journal.pone.0289482)
Supplement: Attachment — Submitted filename: ASC-P1_1st_response_point-by-point_2023-05-03.docx [file pone.0289482.s004.docx]

Response to the Editor's and Reviewer's Comments - Point-by-point

The authors would like to thank for the important and beneficial comments, all of them were considered. The manuscript has been revised accordingly and our responses can be found below (blue text).

# Journal requirements:

1. Please ensure that your manuscript meets PLOS ONE's style requirements, including those for file naming. The PLOS ONE style templates can be found at

https://journals.plos.org/plosone/s/file?id=wjVg/PLOSOne_formatting_sample_main_body.pdf

and

https://journals.plos.org/plosone/s/file?id=ba62/PLOSOne_formatting_sample_title_authors_affiliations.pdf.

The changes have been made as requested and we hope the manuscript now meets your requirements.

2. We note that the grant information you provided in the ‘Funding Information’ and ‘Financial Disclosure’ sections do not match. When you resubmit, please ensure that you provide the correct grant numbers for the awards you received for your study in the ‘Funding Information’ section.

The funding information was reviewed again and added to the cover letter as requested (page 2). Please let us know if we missed discrepancies.

3. Thank you for stating the following financial disclosure:

“We acknowledge co-support from Federal Ministry for Economic Affairs and Energy (grant numbers MG: ZIM 16KN051655, SK: ZIM 16KN051656), the Saxonian State Ministry for Higher Education, Research and the Arts (stipend reference MG: 31004 70 809) and support from Open Access Publishing Fund of Leipzig University. The funders had no role in study design, data collection and analysis, decision to publish, or preparation of the manuscript.

Federal Ministry for Economic Affairs and Energy-ZIM: https://www.zim.de/ZIM/Navigation/DE/Home/home.html

Saxonian State Ministry for Higher Education, Research and the Arts - HTWK Leipzig - Förderlinie N-Promotion 2020: https://gradz.htwk-leipzig.de/

Open Access Publishing Fund of Leipzig University: https://www.ub.uni-leipzig.de/open-science/oa-allgemein/”

Please state what role the funders took in the study. If the funders had no role, please state: "The funders had no role in study design, data collection and analysis, decision to publish, or preparation of the manuscript."

If this statement is not correct you must amend it as needed.

Please include this amended Role of Funder statement in your cover letter; we will change the online submission form on your behalf.

The financial disclosure was reviewed again and added to the cover letter as requested (page 2).

4. Thank you for stating the following in the Acknowledgments Section of your manuscript:

“We acknowledge co-support from Federal Ministry for Economic Affairs and Energy (grant numbers ZIM 16KN051655, ZIM 16KN051656), the Saxonian State Ministry for Higher Education, Research and the Arts (stipend reference 31004 70 809) and support from Open Access Publishing Fund of Leipzig University. The sponsors played no role in the study design, data collection, data analysis, data interpretation, or writing of the manuscript. Special thanks to Prof. Dr. Ing. Volker Slowik and Dr. Ing. Thomas Klink from the Leipzig University of Applied Sciences for their continuous support.”

We note that you have provided funding information that is not currently declared in your Funding Statement. However, funding information should not appear in the Acknowledgments section or other areas of your manuscript. We will only publish funding information present in the Funding Statement section of the online submission form.

Please remove any funding-related text from the manuscript and let us know how you would like to update your Funding Statement. Currently, your Funding Statement reads as follows:

“We acknowledge co-support from Federal Ministry for Economic Affairs and Energy (grant numbers MG: ZIM 16KN051655, SK: ZIM 16KN051656), the Saxonian State Ministry for Higher Education, Research and the Arts (stipend reference MG: 31004 70 809) and support from Open Access Publishing Fund of Leipzig University. The funders had no role in study design, data collection and analysis, decision to publish, or preparation of the manuscript.

Federal Ministry for Economic Affairs and Energy-ZIM: https://www.zim.de/ZIM/Navigation/DE/Home/home.html

Saxonian State Ministry for Higher Education, Research and the Arts - HTWK Leipzig - Förderlinie N-Promotion 2020: https://gradz.htwk-leipzig.de/

Open Access Publishing Fund of Leipzig University: https://www.ub.uni-leipzig.de/open-science/oa-allgemein/”

Please include your amended statements within your cover letter; we will change the online submission form on your behalf.

All funding information was removed from the manuscript as requested and added to the cover letter (page 2). The acknowledgment section was therefore updated in the manuscript.

# Additional editor comments:

1. Lacks Bioethics Committee approval numbers.

There are no bioethics committee approval numbers, since the Institute of Anatomy of Leipzig University has a general institutional approval for the use of postmortem tissues of human body donors as declared in the manuscripts at the beginning of the methods section under preparation acquisition (lines 82-89):

“All tissues originated from the Institute of Anatomy of Leipzig University. While alive, all body donors gave their informed and written consent to the donation of their post-mortem tissues for education and research purposes. Being part of the body donor program regulated by the Saxonian Death and Funeral Act of 1994 (3rd section, paragraph 18, item 8), institutional approval for the use of the post-mortem tissues was obtained from the Institute of Anatomy, Leipzig University. The authors declare that all experiments were performed according to the ethical principles of the Declaration of Helsinki.”

Accordingly, this has already been formulated in a variety of publications, for example:

Hammer, Niels; Glätzner, Juliane; Feja, Christine; Kühne, Christian; Meixensberger, Jürgen; Planitzer, Uwe et al. (2015): Human vagus nerve branching in the cervical region. In: PloS one 10 (2), e0118006. DOI: 10.1371/journal.pone.0118006.

The separate section “Ethics Decleration” has been removed according to the E-Mail from May 3, 2023.

2. Methodology -needs to be described in detail.

More details were added in lines 126-127, 151-172, 185-187, 195-196, 202-209, 215-216, 250-260 and 269-274.

3. Results - structure the presentation of results, with subsections/subsections.

Changed as requested in lines 288 and 313.

4. Discussion -needs to be developed.

Developed and changed as requested in lines 348-357 and 380-391.

# Reviewers’ comments:

No additional comments were received.

# PACE:

While revising your submission, please upload your figure files to the Preflight Analysis and Conversion Engine (PACE) digital diagnostic tool, https://pacev2.apexcovantage.com/. PACE helps ensure that figures meet PLOS requirements. To use PACE, you must first register as a user. Registration is free. Then, login and navigate to the UPLOAD tab, where you will find detailed instructions on how to use the tool. If you encounter any issues or have any questions when using PACE, please email PLOS at figures@plos.org. Please note that Supporting Information files do not need this step.

Uploaded, converted and replaced as requested.

# Additional journal requirements:

1. Please remove your figures/ from within your manuscript file, leaving only the individual TIFF/EPS image files. These will be automatically included in the reviewer’s PDF

Changed as requested in lines 128, 168, 180, 224, 241, 264, 301, 328 and 341.

2. Your ethics statement should only appear in the Methods section of your manuscript. If your ethics statement is written in any section besides the Methods, please move it to the Methods section and delete it from any other section. Please ensure that your ethics statement is included in your manuscript, as the ethics statement entered into the online submission form will not be published alongside your manuscript.

Deleted as requested.
